# Effect of a 6-Week Preseason Training Protocol on Physiological and Muscle Damage Markers in High-Level Female and Male Basketball Players

**DOI:** 10.3390/sports11110229

**Published:** 2023-11-16

**Authors:** Dimitrios Mexis, Tzortzis Nomikos, Nikolaos Mitsopoulos, Nikolaos Kostopoulos

**Affiliations:** 1 School of Physical Education and Sport Science, Department of Physical Education and Sport Science, National and Kapodistrian University of Athens, 17237 Athens, Greece; dimexis@phed.uoa.gr (D.M.); nickmitsopo@phed.uoa.gr (N.M.); 2 School of Health Sciences and Education, Department of Nutrition and Dietetics, Harokopio University, 17676 Athens, Greece; tnomikos@hua.gr

**Keywords:** basketball, preseason training, exercise-induced muscle damage, functional training, plyometric training, performance

## Abstract

This study aimed to investigate the effects of a 6-week preseason functional and plyometric fitness training protocol, on physiological and biochemical markers of performance and exercise-induced muscle damage, and to compare the response of these markers between high-level female and male basketball players. The sample of the study consisted of 19 professional athletes (10 male; 9 female) competing in two different teams. The examined markers were body mass, BMI, fat percentage, speed, acceleration, explosiveness, vertical jumping ability, creatine kinase (CK) and lactate dehydrogenase (LDH). The preseason training period improved speed, acceleration, explosiveness and vertical jumping ability (~1–8%) and led to significant fat percentage reductions in both groups equivalently. CK and LDH increased similarly in both groups, and the percentage increases were higher for CK compared to LDH. Further investigation and a larger sample size are required in order to determine an approach that is more capable of maximizing performance without causing any possible injuries that may be related to muscle damage.

## 1. Introduction

High-level basketball requires specific skills and physical attributes such as explosiveness, strength and jumping ability that, in most cases, are performed under intense and dynamic conditions as athletes move around at high speed or change direction [1,2,3,4]. Additionally, while most skills during training or matches are performed at high intensity and anaerobic metabolism is the main energy source, a certain level of aerobic endurance is just as important to meet energy demands for the whole competitive season [5,6].

As in most team sports, the preseason period in basketball is the most important part of preparation for athletes to endure the challenges of the regular season successfully [7,8]. In recent years, especially at the high level, many basketball and fitness coaches have been trying to discover new ways and new training techniques during the preseason period in order to help their players prepare as best as possible while avoiding any possible injuries [9]. Functional and plyometric training are two of the methods that are currently being used in high-level basketball in order to maximize the physical performance of athletes. Functional training includes a customized combination of exercises for the entire body of the athlete, during which many different joints and muscle groups are used at the same time [9,10]. This method is based on seven basic movements (gait, pull, push, lunge, squat, rotation and hinge), which we all use on a daily basis [9,10]. Plyometric training is one of the most known and effective methods and is used in a multitude of team and individual sports. It is one of the best methods for developing different types of explosive abilities and can be described as any training containing eccentric–concentric muscle activity where there is extended muscle elongation [2,11]. Plyometric exercises are intended to combine the power and speed of movement to promote the development of explosiveness [11,12].

An essential procedure for maximizing the physical performance of athletes at the high level is the assessment of specific physical abilities and analysis of outcomes [13]. The analysis and assessment of these physical abilities are carried out through scientifically acceptable and approved tests that are widely known as the physiological assessment of athletes [13,14,15]. Specifically in basketball, the abilities that are evaluated through the physiological assessment are directly related to game-specific requirements. A lot of sprints up to 20 m and many consecutive jumps characterize the game of basketball. Considering this, it is reasonable that the physiological assessment of basketball athletes will focus more on abilities such as speed, explosiveness and vertical jumping [1,4]. 

Furthermore, high-intensity basketball training can lead to increased levels of muscle damage in basketball athletes [16,17,18], which usually accumulate during the preseason and in-season periods and are sensed by athletes as signs of fatigue, leading to reduced performance and an increased possibility of injuries [19,20]. There are several methods to monitor specific parameters of fatigue that can help coaches to optimize their training regimens, such as the determination of various biochemical markers through blood or saliva samples [21,22], including creatine kinase (CK) and lactate dehydrogenase (LDH) [23].

According to these data, this study aimed to investigate the effects of a 6-week preseason functional and plyometric fitness training protocol on physiological and biochemical markers that are directly related to performance and muscle damage between high-level male and female basketball athletes.

## 2. Materials and Methods

### 2.1. Participants

The total sample of the study consisted of 19 professional athletes (10 male; 9 female) competing in 2 different teams. All athletes of the study were participating in the Greek National A2 Male or Female Division, respectively. The main characteristics of the participants are shown in Table 1. The selection of the sample was made following specific inclusion criteria: (1) high-level professional athletes, (2) ≥18 years old, (3) at least 4 years of basketball experience, and (4) no injuries for at least 3 months before the start of the study. All participants were informed in detail before the start of the study of the purpose, analytical procedures, requirements and benefits of this research and signed the necessary consent documents for their participation. The Bioethics Committee of the School of Physical Education and Sport Science of the National and Kapodistrian University of Athens granted the ethics approval for the current study (1209/16 September 2020), in accordance with the ethical criteria of the Helsinki Declaration.

### 2.2. Study Design

This study was an experimental study, during which the athletes were tested before and after the 6-week preseason period. The intervention program was the independent variable, while the physiological and biochemical parameters that were assessed were the dependent variables. Weight, body mass index (BMI), fat percentage, speed, acceleration, explosiveness and vertical jumping ability were the physiological markers that were evaluated, whilst total creatine kinase (CK) and lactate dehydrogenase (LDH) activities were the examined biochemical markers. Also, it has to be mentioned that in serum samples, total CK activity is determined mainly by the skeletal muscles and the MM isoenzyme is the dominant fraction [24]. In terms of physical training and technical and tactical basketball training, both teams followed the exact same preseason protocol.

### 2.3. Intervention Program

The intervention fitness protocol lasted for the whole 6 weeks of the preseason period for both teams, and was separated in strength and conditioning training units. The strength training units were always executed before the main tactical and technical basketball training units, while the conditioning training units were executed after the warm-up and the stretching of the main tactical and technical basketball training units. Total tactical and technical basketball training units and friendly games were 47; total strength training units were 12 and total conditioning training units were also 12. Exercises, intensity and duration of all the training units were exactly the same for both of the 2 teams. Concerning the strength training, training load for each athlete was calculated using the 1RM for the upper (bench press) and the lower (squat) body part. Also, the exercises that were used for the strength training were based on functional and plyometric training, whilst the exercises for the conditioning training were exclusively basketball-related fitness drills (side steps, turns, back steps, 5, 10 or 20 m sprints, jumping). The number of the strength and conditioning training units, including the intensity and duration that were carried out during the 6 week preseason period are presented in Table 2.

Additionally, in Table 3, we demonstrate the detailed fitness training protocol that took place during the first and the last week of the preseason period.

### 2.4. Biochemical Assessment

The participants arrived at the laboratory of Harokopio University twice: on the morning of the first day of the preseason period and on the morning after the last training day of the preseason period, after 8–12 h of fasting. Blood samples were collected by a trained phlebotomist under the supervision of a doctor. Ten (10) mL of blood was collected in vacutainers without anticoagulant and left at room temperature for 30 min. The tubes were then centrifuged at 1500× *g* for 20 min at a temperature of 4 °C and the supernatant (serum) was collected and aliquoted in Eppendorf tubes. Six (6) mL of blood was also collected in vacutainers with EDTA anticoagulant. These tubes were also centrifuged at 1500× *g* for 10 min at a temperature of 4 °C and the supernatant (plasma) was also collected and aliquoted. Serum CK and LDH activities were analyzed using an automated chemistry analyzer (Konelab 60i, Thermo Fisher Scientific, Waltham, MA, USA) in a private diagnostic center. 

### 2.5. Physiological Assessment

Height was measured with a portable stadiometer (ADE MZ10042, Ade, Hamburg, Germany), while weight was measured with a precision electronic scale (Beurer BF 1000 Super Precision, Beurer, Ulm, Germany). The BMI was calculated through the equation weight (kg) ÷ [height (m)]^2^. The 7-spot (chest, midaxilar, triceps, subscapular, abdominal, suprailiac, thigh) Jackson and Pollock formula was used for the calculation of fat percentage, performed with a precision skinfold caliper (Harpenden Skinfold Caliper, Baty International, Sheffield, UK). Speed, acceleration and explosiveness were assessed through linear sprint tests of 20, 10 and 5 m, respectively, and the time was recorded electronically via photocells (Witty, Microgate, Bolzano, Italy). The first 2 photocells were placed on the start line, and the next 6 photocells were placed 5, 10 and 20 m away from the start line. The participants began to sprint 20 cm before the start line, while the time was recorded from when they passed through the first 2 photocells next to the start line, and recording stopped when they passed through the last 2 photocells, 20 m away. The times held through the photogates were from 5, 10 and 20 m. Finally, vertical jumping ability was assessed through the counter movement jump test (CMJ) with free arms swing, in a portable photocells device (Optojump, Microgate, Bolzano, Italy). Every athlete started the test in a standing position, and after making a deep squat, they tried to jump as high as possible. Also, the athletes had to stretch their arms up during the squat, and to continue the opposite arm movement during the propulsive phase, in order to provide their bodies with a greater boost. All the measurements took place inside the basketball gym and were carried out twice: before the start of the first training of the preseason period and one day after the last training of the preseason period.

### 2.6. Statistical Analysis

Analysis of the data was executed using the SPSS software (version 25.0, IBM Corp., Armonk, NY, USA). Normal distribution of the data was assessed using the Kolmogorov–Smirnov test. When the variables followed parametric distribution, the results were presented as mean ± standard deviation, while in the case of non-parametric distribution, the results were presented as median (25°–75° percentile). The percentage changes of the variables before and after the preseason period were calculated using the equation [(post value − pre value)/pre value] ∗ 100. A *t*-test analysis was used to compare the mean values of each variable for the two groups at the same time point, while a paired *t*-test was used to assess the post-training vs. pre-training differences of the dependent variables for each group separately. In the cases of non-parametric values, the Wilcoxon test and the Wilcoxon signed-rank test were used accordingly. The magnitude of percentage change was assessed using the Cohen’s *d* effect size (ES) model (<0.1 = trivial effect, 0.1–0.3 = small effect, 0.3–0.5 = moderate effect, >0.5 = large effect). The effects of each group on each dependent variable were evaluated using a two-way analysis of variance (ANOVA) with repeated measures across time. The Levene’s homogeneity of variance test was performed before the ANOVA test to assess the equality of variances of the examined variables. Due to multiple comparisons, a post hoc Bonferroni correction was executed in order to correct the type I error and approach the level of significance that was set for the statistical analyses of this study. The correlations between the variables were carried out using the Pearson correlation test for parametric distribution, and the Spearman correlation test for non-parametric distribution. Correlation magnitude was determined according to Cohen’s provided guidelines (*r* = ~0.1 → small effect, ~0.3 → medium effect, ≥0.5 → large effect). The effect size for the ANOVA was evaluated using the η_p_^2^ model, while all statistical differences between the mean values of the variables were calculated using a *p* = 0.05 significance level and a 95% confidence interval (CI).

## 3. Results

### 3.1. Descriptive Characteristics

The sample of the study consisted of two teams (men, women), whose descriptive characteristics as recorded before the study are presented in Table 1. As it was expected, the female group was characterized by a lower height and weight and a higher fat percentage compared to male participants. No significant differences were observed for age and BMI between the two teams before the start of the study.

### 3.2. Effect of Preseason Training on Body Composition

The preseason training period leads to mild fat percentage reductions while it does not affect weight or BMI in both the male and female groups. Males seem to be better benefited compared to females, in terms of fat percentage reduction (Table 4). 

### 3.3. Effect of Preseason Training on Performance Indices

Preseason training favorably affected physiological indices of performance such as speed—20 m, acceleration—10 m, explosiveness—5 m and vertical jumping ability (Table 5) in both males and females basketball players. The observed improvements ranged from a ~1% reduction in sprint times to an ~8% increase in vertical jumping ability. Similar improvements (in terms of percentage change) were observed between male and female athletes. 

### 3.4. Effect of Preseason Training on CK and LDH

Male athletes showed greater values of CK and LDH before the start of the preseason period compared to females. Preseason training increased the activities of CK and LDH in both groups. The percentage increases were higher for CK compared to LDH. The percentage changes did not differ significantly between groups, although there was a trend for higher percent increases in CK in female athletes and LDH in male athletes (Table 6).

### 3.5. Correlations

In an attempt to investigate possible correlations between the percentage changes of the measured variables, we proceeded to conduct a correlation analysis. Expected correlations were observed, such as between speed and acceleration of both of the teams (men: *r* = 0.71, *p* = 0.021, women: *r* = 0.938, *p* < 0.001). Additionally, the women’s team also showed positive correlations between weight and fat percentage (*r* = 0.897, *p* < 0.001), and between CK and LDH (*r* = 0.913, *p* < 0.001) (Table 7).

## 4. Discussion

This was the first study that attempted to investigate gender differences directly related to performance and muscle damage between high-level male and female basketball athletes. Also, it was the first study that attempted to evaluate and correlate physiological and biochemical indices before and after the preseason period, since speed, acceleration, explosiveness and vertical jumping ability are essential attributes of modern basketball, along with the determination of CK and LDH, both of which are widely acknowledged as two of the best methods to identify muscle damage. The results showed significant reductions in fat percentage and improvements in every physiological marker, but no differences were found between the two groups. CK and LDH also similarly increased in both groups. 

In terms of height, weight and fat percentage, we demonstrated significant differences between the two teams before the beginning and after the end of the preseason period. This was expected, since men have a larger body size and higher muscle-to-fat mass ratio compared to women [25]. Also, women have a naturally higher percentage of fat mass compared to men [26]. On the other hand, there were no significant differences in age and BMI between the two teams. Body mass index (BMI) is the same for adult men and women, so it was reasonable that we did not find any differences between the two teams. The fact that both groups showed similar age and BMI conferred a better reliability of the study.

Body fat was reduced after the preseason period in both teams. This outcome comes in agreement with previous studies that demonstrated a significant loss of fat in female (−3.1%) and male (−3.49%) basketball players after 8 and 6 weeks of preseason training [27,28]. Finally, this significant decrease in fat percentage in both teams did not lead to any significant alterations in body weight. This finding suggests that all the players lost a significant amount of body fat but they also gained more muscle mass; that is why their body weight remained on levels similar to those before the preseason training period [27].

The men’s team showed significantly greater values in all four physiological markers (speed, acceleration, explosiveness, vertical jumping ability) compared to the women’s team before and after the preseason period. These differences are mainly attributed to gender dissimilarities [29], such as the increased lean muscle mass of male athletes. Also, the significant improvements in both teams after the preseason period in the four physiological markers that were assessed have been validated by many previous studies. The improvement of the above markers implies that the training protocol applied in our study was effective to improve the performance of high-level male and female basketball players. More specifically, vertical jumping ability was significantly improved after 3 (8.08%), 8 (~8.77%) and 10 weeks (7–12.2%) of preseason plyometric and block periodization training in female and male elite basketball athletes [30,31,32]. Sprint performance was also significantly improved after 3 (1.55%), 4 (1.49%) and 6 (~1.25–1.65%) weeks of preseason training in female and male elite basketball athletes, through 10 and 20 m sprinting tests [30,31,33]. Some other studies demonstrated a significant effect of plyometric training on explosiveness through 5 m sprinting tests on young basketball players (~4%) [12,34]. Finally, no differences were found between the two teams concerning the percentage changes of the physiological markers, meaning that both teams showed similar improvements.

In a practical point of view, an improvement in speed at 0.035 s (males) and 0.032 s (females) offers the players a sufficiently larger amount of time to perform sprints or specific basketball moves faster than they had before. If we consider that a single sprint in basketball from 5 m up to 20 m can last ~1–5 s [5,21,35], the improvements that we found in speed, acceleration and explosiveness show that the players will be more efficient to perform basketball-specific moves after executing our training regimen, thus enhancing significantly their performance. Additionally, a basketball player can execute an average of 46 jumps during an official basketball game, while vertical jump height ranges from ~35 cm to ~75 cm in professional male and female basketball athletes [5,21,36,37]. In our study, vertical jump was significantly improved by 3.4 cm (males) and 2.3 cm (females). Considering the previous data, the ability to jump higher in basketball is very crucial, and an improvement of 8.72% and 6.75% for male and female basketball athletes, respectively, is more than adequate to enhance vertical jumping ability.

Male athletes showed greater values of baseline CK and LDH compared to females before the start of the preseason period. Increased muscle mass and elevated levels of testosterone have been previously correlated with elevated CK levels [24,38], which may explain why male athletes appeared to have higher CK and LDH activities compared to females [39]. Also, CK and LDH levels were significantly higher after the preseason period for both of the teams, a finding that comes in agreement with previous studies that demonstrated a significant increase in CK and LDH levels after 2 h of high-intensity basketball training (208.41%), or after a single resistance training unit (6–12%) [40,41]. Significant increases have been also observed after an official basketball match (~125–160%) [16,42], or during a competitive basketball season [43,44]. There is also a trend for a higher percent increase in LDH activity in the men’s team compared to the women’s team after the end of the preseason period. This trend was not observed for the CK values. Sewright et al. [45] had also found higher responses of CK in male subjects compared to females after 4 days of eccentric exercise (55.8%). These differences could be hormone dependent, and they could be linked with estrogen and increased levels of circulating estradiol in the female population, since it has been previously reported that estrogen may affect CK levels in women after exercise through maintaining post-exercise membrane stability [24,46,47].

Taking into consideration the normal reference values for CK and LDH activities, our findings show that the increase is not too extensive. Nevertheless, similar values of CK and LDH have been previously associated with bone and muscle injuries [48,49], so it is crucial that we keep examining the underlying mechanisms that cause these impairments, and attempt to better understand the role of CK and LDH in these procedures. Furthermore, CK and LDH appear to be adequate indicators in acquiring an appropriate perspective on recovery strategies and underlying mechanisms [50]. Due to the simplicity of their measurements, CK and LDH are still the most common biochemical markers of exercise-induced muscle damage, despite the fact that they do not always completely reflect the extend of the damage. Therefore, the interpretation of their changes after a preseason training period should be critically evaluated, also in terms of adjusting the recovery strategies of the athletes [51]. In our study, the observed changes indicate a mild muscle damage, which is expected after the preseason training, with no apparent clinical and physiological effect, in both male and female athletes.

The improvement in speed was positively correlated with the improvement of acceleration in both teams. This finding suggests that after 10 m of linear sprinting and up to 20 m, the performance of all athletes was significantly better after the preseason period. The study by Shalfawi et al. [52] also demonstrated a strong relationship between 10 and 20 m linear sprinting tests in male basketball players. This result is critical, if we consider that basketball players run distances between 10 and 20 m very often during training or official matches [53]. Additionally, a significant positive correlation was found between the weight and the fat percentage reduction in female athletes. Even a mild loss of body weight can very often lead to fat percentage reduction and vice versa, this procedure is enhanced when it comes to professional athletes with heavy training protocols [27,54]. Finally, a strong correlation between the CK and the LDH was noticed in female athletes. These markers displayed a similar increase after the preseason period for both teams, and confirmed an aftereffect muscle damage in the participants [55]. This finding comes in agreement with the study of Khajehlandi and Janbozorgi [41], that demonstrated a similar increase in CK and LDH enzymes in female basketball athletes after only a single session of exercise. Finally, no significant correlations were found between the examined markers and the position (center, forward, guard) or the basic characteristics (age, height, weight) among athletes.

In conclusion, our study led to significant physical fitness improvements both on male and female basketball athletes. Nevertheless, these improvements induced a significant increase in CK and LDH on both teams. Also, our research had some essential limitations, such as a lack of a control group and a small sample of athletes. Future studies should try to use a larger sample size, examine a longer preseason training period or attempt to diversify these kinds of protocols and achieve a better balance between enhanced performance and induced muscle damage.

## 5. Practical Applications

A 6-week preseason basketball training protocol, based on functional and plyometric fitness training, can lead to significant improvements in speed, acceleration, explosiveness and vertical jumping ability, both in male and on female high-level basketball athletes. Additionally, this specific kind of training can significantly reduce the percentage of body fat on every athlete. Basketball coaches and trainers must consider these results and decide which approach is more capable to maximize the performance of their athletes, without causing any possible injuries that may be related to extended muscle damage.

## Figures and Tables

**Table 1:** Descriptive characteristics.

	Men	Women	*p*
**Age** (years)	25.1 ± 6.2	22.7 ± 5.9	0.396
**Height** (cm)	195 ± 0.06	171 ± 0.04	<0.001 *
**Weight** (kg)	91.8 ± 10.4	71.2 ± 9.5	<0.001 *
**ΒΜΙ** (kg/m^2^)	24.0 ± 1.4	24.3 ± 3.2	0.748
**Fat Perc** (%)	14.3 ± 3.2	23.4 ± 3.5	<0.001 *

* Significant differences between the two teams.

**Table 2:** Strength and conditioning training units.

1st Week	2nd Week	3rd Week	4th Week	5th Week	6th Week
2 strength (60′ medium intensity)—1 conditioning (30′ low intensity)	2 strength (60′ medium intensity)—2 conditioning (30′ medium intensity)	3 strength (50′ high intensity)—2 conditioning (40′ medium intensity and 30′ high intensity)	2 strength (50′ high intensity)—3 conditioning (30′ high intensity × 2 and 40′ medium intensity)	2 strength (50′ high to medium intensity)—2 conditioning (30′ high to medium intensity)	1 strength (50′ medium to low intensity)—2 conditioning (20′ medium to low intensity)

**Table 3:** First and sixth week analytical fitness protocol.

	1st Week	6th Week
Monday	(1) Fitball planks 3 × 40 s. (2) Forward lunges on bosu 2 × 15 for each leg. (3) TRX back row 3 × 15. (4) Arm biceps with exercise band 3 × 20. (5) Fitball pullovers with dumbbell 3 × 12. (6) Smith squats 3 × 12-10-8. (7) Push-ups 3 × 12. (8) Shoulders press with dumbbells 3 × 10. (9) Abs exercises (upper–lower side) 3 × 15. (10) Back exercises 3 × 15. + Basketball training	(1) Ladder drills 5′. (2) Nebraska agility cone drill 45″ × 5. (3) Wheel bag barrier drill 4 sets. (4) 4 corner cone drill 40″ × 4. + Basketball training
Tuesday	Basketball training (morning) + Basketball training (afternoon)	(1) Wall squats 40″ × 3. (2) Fitball planks 40″ × 3. (3) TRX back row 3 × 15. (4) Single-leg RDL with dumbbells 3 × 10. (5) Dumbbell chest press on a fitball 3 × 12. (6) Bulgarian split squat with dumbbells 3 × 10. (7) Arnold press exercise 3 × 12-10-8. (8) Abs exercises (upper-lower side) 3 × 15. (9) Back exercises 3 × 15. + Basketball training
Wednesday	(1) Ladder drills 5′. (2) Cone ladder drill 5-10-5 50″ × 5. (3) 4 corner cone drill 45″ × 5. (4) Tap bag barrier drill 5 sets. (5) Change of pace cone drill 40″ × 4. + Basketball training	Basketball—friendly game
Thursday	Basketball training	Basketball training (morning) + Basketball training (afternoon)
Friday	Basketball training (morning) + Basketball training (afternoon)	(1) Ladder drills 5′. (2) Sprint and shuffle cone drill 40″ × 4. (3) Crossover and step barrier drill 4 sets. (4) V cone drill 35″ × 4. + Basketball training
Saturday	(1) Single-leg balance exercises on bosu 40″ × 3. (2) Shoulder external and internal rotation with exercise band 3 × 15 for each arm. (3) Bosu squats 3 × 15. (4) Closed grip push-ups 3 × 12. (5) TRX front shoulder raises 3 × 15. (6) Bulgarian split squat with dumbbells 3 × 12. (7) Bench press 3 × 12-10-8. (8) Single-arm dumbbell back row 3 × 12. (9) Abs exercises (upper-lower side) 3 × 15. (10) Back exercises 3 × 15. + Basketball training	Basketball training
Sunday	Rest	1st game of the regular season

**Table 4:** Effects on somatometric characteristics.

		Before	After	Percentage Change *(p)*	ES (95% CI)	T *p* (η_p_^2^)	TG *p* (η_p_^2^)	G *p* (η_p_^2^)
**Weight** (kg)	**Men**	91.8 ± 10.4	91.6 ± 10.6	−0.18 ± 1.55% (0.748)	−0.01 (−1.25, 1.22)	0.297 (0.064)	0.623 (0.015)	<0.001 ^†^ (0.547)
	**Women**	71.2 ± 9.5	70.8 ± 9.1	−0.50 ± 0.91% (0.101)	−0.04 (−1.35, 1.26)			
	***p***	<0.001 *	<0.001 *	0.605				
**BMI** (kg/m^2^)	**Men**	24.0 ± 1.4	24.0 ± 1.4	0.09 ± 1.75% (0.882)	0.01 (−1.22, 1.25)	0.426 (0.038)	0.297 (0.064)	0.800 (0.004)
	**Women**	24.3 ± 3.2	24.2 ± 3	−0.53 ± 0.8% (0.063)	−0.03 (−1.33, 1.27)			
	***p***	0.748	0.855	0.337				
**Fat Perc** (%)	**Men**	14.3 ± 3.2	13.8 ± 3.1	−3.82 ± 4.02% (0.013) ^#^	−0.16 (−1.4, 1.08)	<0.001 ^†^ (0.484)	0.270 (0.071)	<0.001 ^†^ (0.687)
	**Women**	23.43 ± 3.5	23.13 ± 3.3	−1.18 ± 1.32% (0.024) ^#^	−0.09 (−1.39, 1.21)			
	***p***	<0.001 *	<0.001 *	0.077				

T, Time; TG, Time × Group; G, Group. * Significant differences between the two teams before and after the preseason period. ^#^ Significant differences before and after the preseason period for each team separately. ^†^ Significant interaction between the two teams (two-way repeated measures ANOVA).

**Table 5:** Effects on physiological indices.

		Before	After	Percentage Change *(p)*	ES (95% CI)	T *p* (η_p_^2^)	TG *p* (η_p_^2^)	G *p* (η_p_^2^)
**Speed** **20 m** **(s)**	**Men**	3.333 ± 0.3	3.298 ± 0.3	−1.04 ± 0.87% (0.006) ^#^	−0.12 (−1.36, 1.12)	<0.001 ^†^ (0.512)	0.807 (0.004)	0.019 ^†^ (0.284)
	**Women**	3.67 ± 0.26	3.638 ± 0.25	−0.82 ± 1.02% (0.038) ^#^	−0.13 (−1.43, 1.18)			
	***p***	0.021 *	0.018 *	0.620				
**Acceleration** **10 m** **(s)**	**Men**	2.161 ± 0.1	2.142 ± 0.1	−0.87 ± 0.73% (0.004) ^#^	−0.19 (−1.43, 1.05)	<0.001 ^†^ (0.579)	0.636 (0.013)	0.017 ^†^ (0.292)
	**Women**	2.3 ± 0.12	2.284 ± 0.12	−0.66 ± 0.64% (0.015) ^#^	−0.13 (−1.44, 1.18)			
	***p***	0.019 *	0.015 *	0.518				
**Explosiveness** **5 m** **(s)**	**Men**	1.387 ± 0.03	1.373 ± 0.03	−1 ± 0.78% (0.003) ^#^	−0.47 (−1.72, 0.79)	<0.001 ^†^ (0.692)	0.487 (0.029)	0.021 ^†^ (0.276)
	**Women**	1.452 ± 0.07	1.441 ± 0.07	−0.76 ± 0.42% (<0.001) ^#^	−0.16 (−1.47, 1.15)			
	***p***	0.024 *	0.018 *	0.443				
**Vertical Jumping Ability** **(cm)**	**Men**	42.43 ± 6.27	45.85 ± 5.04	8.72 ± 5.9% (<0.001) ^#^	0.6 (−0.67, 1.87)	<0.001 ^†^ (0.772)	0.147 (0.120)	<0.001 ^†^ (0.482)
	**Women**	34.59 ± 3.26	36.87 ± 3.04	6.75 ± 4.3% (<0.001) ^#^	0.72 (−0.63, 2.07)			
	***p***	0.004 *	<0.001 *	0.442				

T, Time; TG, Time × Group; G, Group. * Significant differences between the two teams before and after the preseason period. ^#^ Significant differences before and after the preseason period for each team separately. ^†^ Significant interaction between the two teams (two-way repeated measures ANOVA).

**Table 6:** Effects on biochemical indices.

		Before	After	Percentage Change *(p)*	ES (95% CI)	T *p* (η_p_^2^)	TG *p* (η_p_^2^)	G *p* (η_p_^2^)
**CK** **(U/L)**	**Men**	123.7 ± 40.02	207.5 ± 28.74	77.28 ± 42.5% (<0.001) ^#^	2.4 (0.78, 4.03)	<0.001 ^†^ (0.686)	0.948 (0.000)	0.007 ^†^ (0.357)
	**Women**	77.11 ± 10.86	159.11 ± 75.43	112.82 ± 126.94% (0.013) ^#^	1.52 (0.04, 3)			
	***p***	0.004 *	0.076	0.414				
**LDH** **(U/L)**	**Men**	165.6 ± 33.54	260.1 ± 41.5	62.23 ± 39.18% (<0.001) ^#^	2.5 (0.85, 4.16)	<0.001 ^†^ (0.688)	0.039 ^†^ (0.228)	0.002 ^†^ (0.456)
	**Women**	134.22 ± 27.76	178.11 ± 56.07	32.67 ± 36.43% (0.020) ^#^	0.99 (−0.39, 2.37)			
	***p***	0.041 *	0.002 *	0.108				

T, Time; TG, Time × Group; G, Group. * Significant differences between the two teams before and after the preseason period. ^#^ Significant differences before and after the preseason period for each team separately. ^†^ Significant interaction between the two teams (two-way repeated measures ANOVA).

**Table 7:** Correlations between % changes of measured variables.

	Variables *r* Correlation (*p* Value)	*r* Magnitude
**Men (n = 10)**	Speed (20 m) ↓—Acceleration (10 m) ↓ 0.71 (0.021)	Large
**Women (n = 9)**	Weight ↓—Fat % ↓ 0.897 (<0.001)	Large
	CK ↑—LDH ↑ 0.913 (<0.001)	Large
	Speed (20 m) ↓—Acceleration (10 m) ↓ 0.938 (<0.001)	Large

↓ Significant decrease. ↑ Significant increase.

## Data Availability

The data presented in this study are only available upon request from the corresponding author.
